# Renal protective effects of astragalus root in rat models of chronic kidney disease

**DOI:** 10.1007/s10157-023-02356-8

**Published:** 2023-05-04

**Authors:** Shunsuke Goto, Hideki Fujii, Kentaro Watanabe, Mao Shimizu, Hidehisa Okamoto, Kazuo Sakamoto, Keiji Kono, Shinichi Nishi

**Affiliations:** Division of Nephrology and Kidney Center, Kobe University Graduate School of Medicine, 7-5-2 Kusunoki-Cho, Chuo-ku, Kobe, 650-0017 Japan

**Keywords:** Astragalus, Chronic kidney disease, Oxidative stress, Renin-angiotensin system

## Abstract

**Background:**

Astragalus root is a commonly used herb in traditional Chinese medicine. Although renoprotective effects have been reported in some clinical and experimental studies, the details remain unknown.

**Methods:**

We used 5/6 nephrectomized rats as chronic kidney disease (CKD) models. At 10 weeks, they were divided into four groups, namely, CKD, low-dose astragalus (AR400), high-dose astragalus (AR800), and sham groups. At 14 weeks, they were sacrificed for the evaluation of blood, urine, mRNA expression in the kidney, and renal histopathology.

**Results:**

Kidney dysfunction was significantly improved following astragalus administration (creatinine clearance: sham group; 3.8 ± 0.3 mL/min, CKD group; 1.5 ± 0.1 mL/min, AR400 group; 2.5 ± 0.3 mL/min, AR800 group; 2.7 ± 0.1 mL/min). Blood pressure, urinary albumin, and urinary NGAL levels were significantly lower in the astragalus-treated groups than those in the CKD group. Excretion of urinary 8-OHdG, an oxidative stress marker, and intrarenal oxidative stress were lower in the astragalus-treated groups than those in the CKD group. Furthermore, the mRNA expression of NADPH p22 phox, NADPH p47 phox, Nox4, renin, angiotensin II type 1 receptor, and angiotensinogen in the kidney was lower in the astragalus-treated groups compared with the CKD group.

**Conclusion:**

This study suggests that astragalus root slowed CKD progression, possibly through the suppression of oxidative stress and the renin–angiotensin system.

## Introduction

Chronic kidney disease (CKD) is a major public health issue, with an estimated global prevalence of 10% [1]. The development of CKD stages is an independent risk factor for mortality and cardiovascular events, and these risks are more than four times higher in patients with advanced CKD stages than in the population with normal kidney function [2]. In addition, patients who received renal replacement therapy have a lower quality of life [3] and restricted daily activity because dialysis takes a certain amount of time. Therefore, to prevent CKD progression, various clinical strategies, including the control of blood pressure, dietary treatment, use of renin–angiotensin system (RAS) inhibitors, and use of sodium–glucose cotransporter-2 inhibitors are recommended. However, some patients, in whom these treatments are ineffective, reach a state of end-stage kidney disease [4].

Astragalus root is a commonly used herb in traditional Chinese medicine. It has been reported to have renoprotective effects in some clinical and animal studies [5, 6] and has been prescribed for patients with CKD in Japan and China [6, 7] However, the pathophysiological mechanism has not been completely elucidated.

Oxidative stress has been reported to play a role in CKD progression [8]. In previous studies, we reported that some medications with anti-oxidative effects could ameliorate kidney injury in animal models of CKD [9–12]. Because astragalus root has been reported to exhibit anti-oxidative effects, we speculated that it is a mechanism of its renoprotective effect.

RAS is also a crucial factor in CKD progression. Since numerous studies have demonstrated that RAS blockade could slow down CKD progression, many clinical guidelines recommend or suggest using RAS inhibitors as the first-line therapy for patients with hypertension, CKD, and proteinuria [13]. Aqueous extract of astragalus root has been reported to inhibit angiotensin-converting enzymes (ACE) in vitro and in hypertensive rats [14]. Another study has shown that total flavonoids extracted from astragalus seed decreased plasma angiotensin II levels in hypertensive rats [15]. However, since several papers did not show that astragalus inhibited RAS [16–18], it is controversial whether astragalus has a beneficial effect on RAS.

Thus, in this study, we examined the renoprotective effects and their mechanism, including oxidative stress and RAS, of astragalus root using 5/6 nephrectomized rats.

## Material and methods

### Animals and experimental protocol

Male Sprague–Dawley rats were obtained from CLEA Japan Inc. (Tokyo, Japan). The rats were housed with ad libitum food and water supply in a light- and temperature-controlled environment. CKD was induced by 5/6 nephrectomy. At seven weeks of age, the rats underwent a two-thirds nephrectomy of the left kidney, and one week later, the right kidney was excised. Astragalus root (Tsumura & Co, Tokyo, Japan) was administered orally at a low dose of 400 mg/kg/day or a high dose of 800 mg/kg/day for four weeks from 10 weeks of age. The doses of astragalus root were determined based on the formula for dose translation as follows; rat dose (mg/kg) = human dose (mg/kg) × 37/6 [19]. The human dose of astragalus root for chronic kidney disease was 5,000 mg/body/day which was about 83 mg/kg/day assuming that the body weight was 60 kg [20]. Therefore, the rat equivalent dose of astragalus root calculated by the formula was about 514 mg/kg/day. Based on the rat equivalent dose, we determined the doses of astragalus root in the study. At 14 weeks of age, the rats were sacrificed under sodium pentobarbital anesthesia. Twenty-four-hour urine samples were collected using a metabolic cage at 10 and 14 weeks of age. We collected blood samples from the jugular vein at 10 weeks of age and from the left ventricle at sacrifice. The kidneys were removed for immunohistochemical analysis and RNA extraction.

This study was conducted in strict accordance with the recommendation in the Guide for the Care and Use of Laboratory Animals of the National Institutes of Health. The Animal Experiment Facility Ethics Committee approved the protocol (Permit No.: 170301). All surgeries were conducted under anesthesia with intraperitoneally administered medetomidine (0.375 mg/kg), midazolam (2 mg/kg), and butorphanol (2.5 mg/kg). Following the ARRIVE guidelines for reporting experiments involving animals, all efforts were made to minimize suffering.

### Blood and urine measurement

Blood samples were centrifuged for 5 min at 3000 rpm and were stored at − 80 °C until analysis. Serum creatinine, blood urea nitrogen, albumin, total cholesterol, calcium, phosphorus, and glucose levels were measured using a Fuji Dri-Chem 3500 (Fujifilm Japan, Tokyo, Japan). Urinary albumin, creatinine, and neutrophil gelatinase-associated lipocalin (NGAL) levels were measured using an enzyme-linked immunosorbent assay (ELISA) kit (albumin: Nephrat, Exocell, Philadelphia, PA, USA; creatinine: Creatinine Assay Kit, Cayman Chemical, Ann Arbor, MI, USA; NGAL: Lipocain-2 Rat ELISA Kit, Abcam, Cambridge, MA, USA). The urinary 8-hydroxydeoxyguanosine (8-OHdG), a sensitive indicator of oxidative DNA damage, was determined using a sandwich ELISA kit (Japan Institute for Control of Aging, Shizuoka, Japan). Serum aldosterone levels were measured using an Aldosterone ELISA kit (Abcam, Cambridge, UK).

### Blood pressure measurement

Blood pressure was measured by tail-cuff plethysmography (Model MK-2000; Muromachi Kikai Co. Ltd, Tokyo, Japan). To reduce the possibility of stress artifacts, we measured blood pressure 15 min after placing rats in the equipment. The average of 10 measurements was adopted as the value of blood pressure. Blood pressure measurement was performed at 10, 12, and 14 weeks of age.

### Immunohistochemical analysis of oxidative stress and histomorphological analysis in kidney tissue

Kidneys were fixed with a 10% formalin-neutral buffered solution and embedded in paraffin. Then, tissue samples were sliced into 3-µm sections and stained with anti-8-OHdG antibodies (Japan Institute for the Control of Aging, Shizuoka, Japan). Next, we calculated the mean number of 8-OHdG-positive cells in glomeruli per one microscopic field and the ratio of 8-OHdG-positive tubular cells to 8-OHdG-negative tubular cells. For the assessment, we counted the positive cells in 20 random microscopic fields.

For histomorphological analysis, tissue samples embedded in paraffin were sliced into 3-µm sections and stained with periodic acid-Shiff (PAS) and Masson’s trichrome. Glomerular volume was evaluated in PAS-stained tissue and the severity of interstitial fibrosis was evaluated in Masson’s trichrome-stained tissue. The evaluation was performed as described previously [21–23]. In brief, we measured the glomerular tuft area in 30 randomly selected glomeruli per sample and calculated glomerular volume from the glomerular tuft area. For the severity of interstitial fibrosis, we graded sections from the cortex of each kidney as follows: 0, no evidence of interstitial fibrosis; 1, less than 10% involvement; 2, 10–25% involvement; 3, 25–50% involvement; 4, 50–75% involvement; 5, more than 75% involvement. We evaluated interstitial fibrosis in 20 random fields per section.

### RNA extraction and real-time polymerase chain reaction (PCR)

Total RNA was extracted from kidney samples using the ISOGEN kit (Wako Pure Chemical Industries, Osaka, Japan) following the manufacturer’s instructions, and total RNA was reverse transcribed with the ReveTra ACE qPCR RT kit (TOYOBO Co., Ltd., Osaka, Japan). Quantitative PCR was performed using the Light Cycler 350 s Real-Time PCR System (Roche-Diagnostics, Mannheim, Germany) with the SYBR Green Assay with Thunderbird SYBR qPCR Mix (TOYOBO Co., Ltd., Osaka, Japan) according to the manufacturer’s protocol. The analysis was performed using the second derivative maximum method of the LightCycler software (version 4.0; Roche). RNA expression of each gene was corrected for β-actin expression. The following primers were used: rat NADPH p22 phox (5′-GGTGAGCAGTGGACTCCCATT-3′, 5′-TGGTAGGTGGCTGCTTGATG-3′), rat NADPH p47 phox (5′-GTGAAGCCATCGAGGTCATTC-3′, 5′-CCCGCGGCTTCTAATCTGT-3′), rat NADPH oxidase 4 (Nox4; 5′-GAACCCAAGTTCCAAGCTCA-3′, 5′-GCACAAAGGTCCAGAAATCC-3′), rat renin (5′-CTGTGCATACTGGCTCTCCA -3′, 5′-GGCTTGGCCTAAAACTAGGG-3′), rat Angiotensin II type I Receptor (AT1R) (5′-CTCAAGCCTGTCTACGAAAATGAG-3′, 5′-GTGAATGGTCCTTTGGTCGT-3′), rat ACE (5′-TGCCTAGATCCCAAGGTGACTTTGA-3′, 5′-CAACTTCATGGCATCTGCCAGCA-3′), rat angiotensinogen (5′-TTGTGTGAGGAGGGCTGTAT-3′, 5′-TGCTGAGAGTGTAGGTCCTG-3′), rat β-Actin (5′-TGACAGGATGCAGAAGGAGA-3′, 5′-TAGAGCCACCAATCCACACA-3′).

### Statistical analysis

Values are presented as means ± standard error of the mean. For comparison among the four groups, one-way ANOVA followed by the Tukey–Kramer test was used. A value of p < 0.05 was considered statistically significant. Statistical analyses were performed using the Stata/MP 14.2 software for Windows (Stata, College Station, TX, USA).

## Results

### Animal characteristics and changes in blood pressure and kidney parameters

**Fig. 1:**
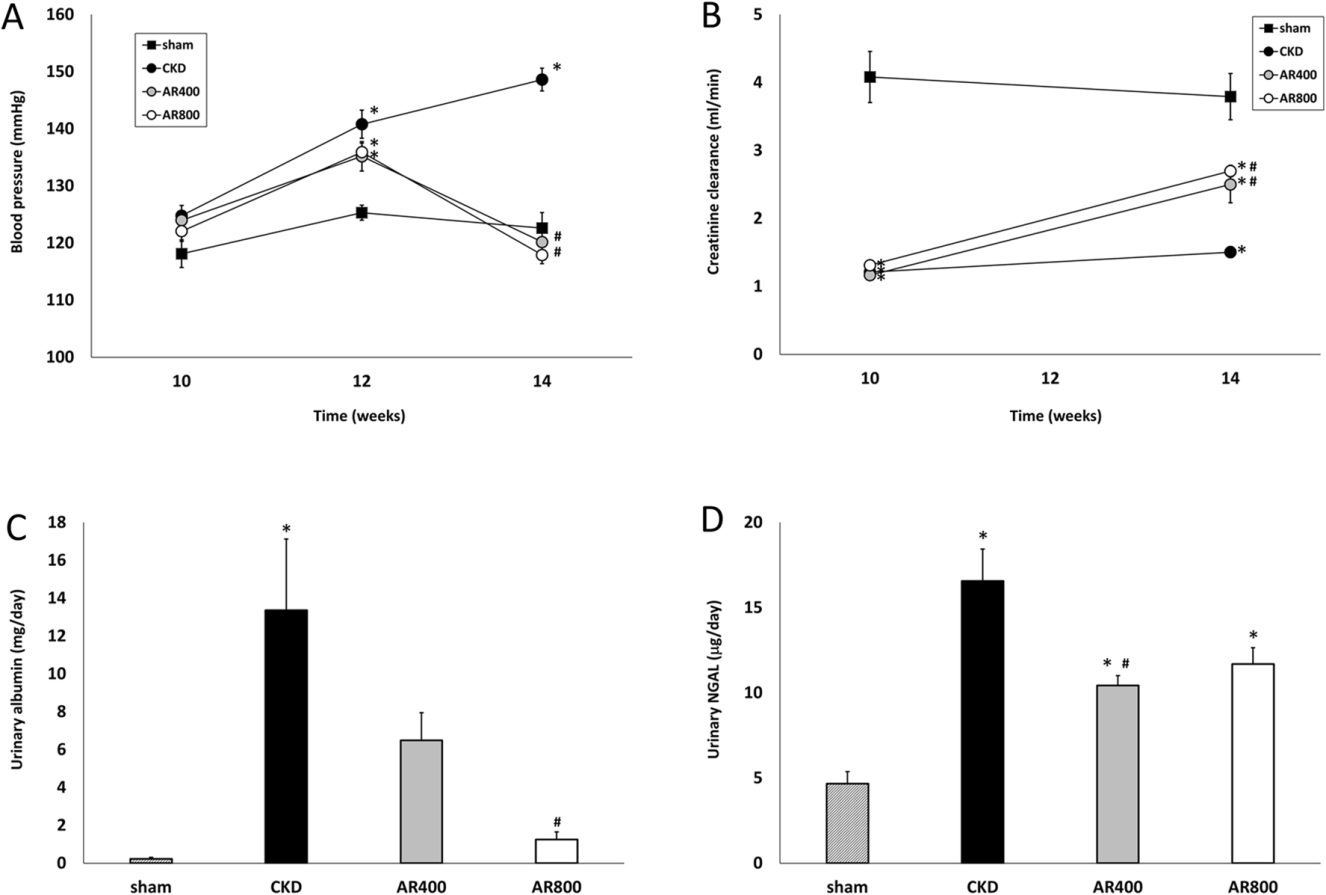
**A** Change in systolic blood pressure, **B** creatinine clearance, **C** urinary albumin levels, and (**D**) urinary NGAL at 14 weeks of age. *P < 0.05 *vs*. the sham group, ^#^*P* < 0.05 *vs*. the CKD group

**Fig. 2:**
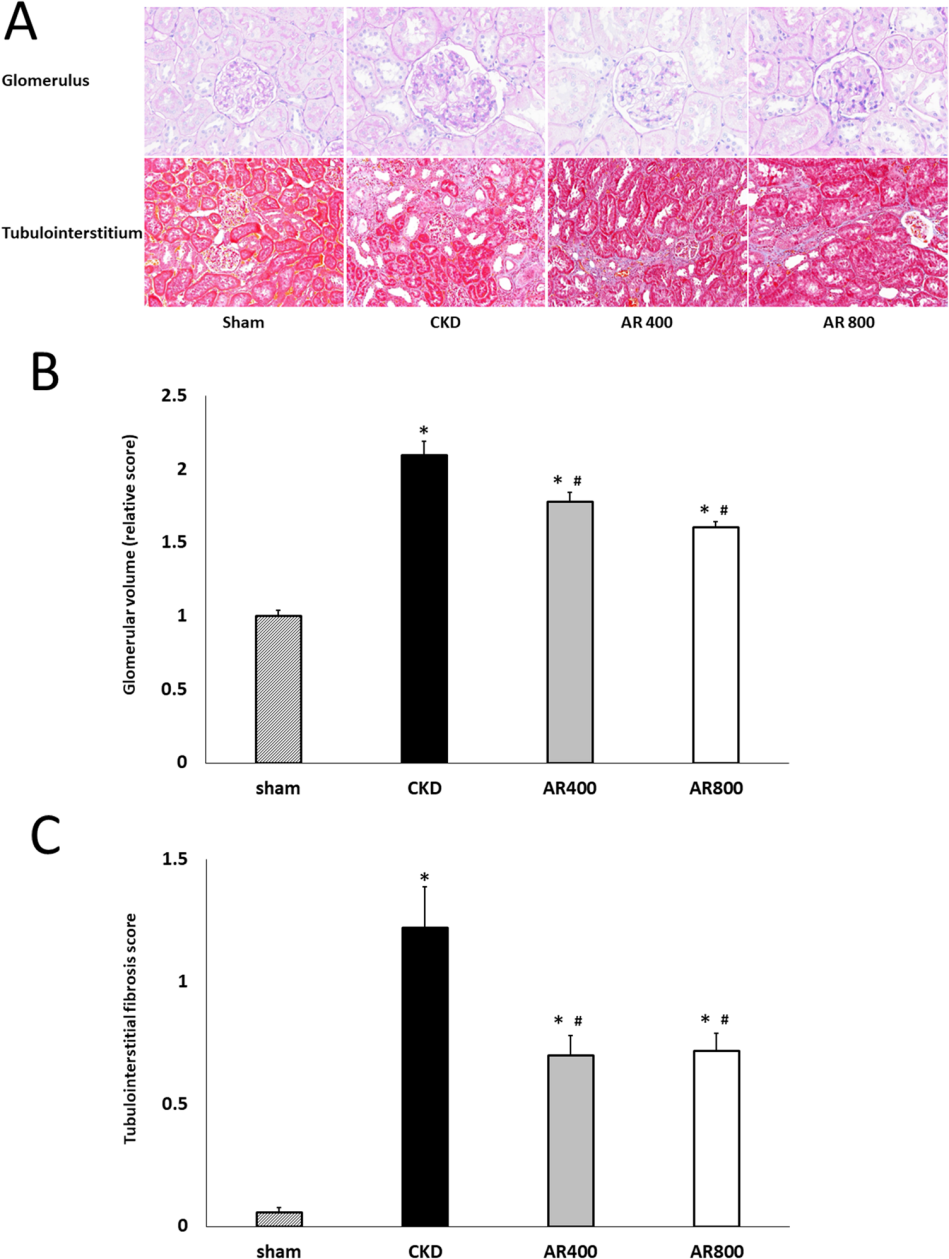
Assessment of renal pathology. **A** Representative images of glomerulus and tubulointerstitium. **B** Glomerular volume. **C** Tubulointerstitial fibrosis

We divided the rats into the following four groups: sham-operated rats (sham, n = 10), CKD rats (CKD, n = 10), CKD rats treated with 400-mg/kg/day astragalus root (AR400, n = 11), and CKD rats treated with 800-mg/kg/day astragalus root (AR800, n = 12). Table 1 shows the animal characteristics of this study population at baseline (10 weeks of age). Kidney function at baseline did not differ between the CKD and astragalus groups. Body weight, blood pressure, serum albumin, total cholesterol, calcium, phosphorus, and glucose levels were comparable among the groups. Urinary protein levels did not increase in the CKD and astragalus groups. The change in systemic blood pressure is indicated in Fig. 1A. Although there was no significant difference in blood pressure at 12 weeks of age between the CKD and astragalus groups, blood pressure at 14 weeks of age in the AR400 and AR800 groups was significantly lower than in the CKD group. Creatinine clearance at 10 and 14 weeks of age is shown in Fig. 1B. Creatinine clearance was recovered in the AR400 and AR800 groups. Urinary albumin levels were higher in the CKD group than in the sham group, those in the AR400 group tended to be lower than those in the CKD group, and those in the AR800 group were significantly lower than those in the CKD group (Fig. 1C). Urinary NGAL levels were also higher in the CKD group than in the sham group, those in the AR800 group tended to be lower than those in the CKD group, and those in the AR400 group were significantly lower than those in the CKD group (Fig. 1D). Other animal characteristics at sacrifice were shown in Table 2. Urine volume, residual kidney weight, and urinary protein levels in the CKD group were higher than that in the sham group. Among these characteristics, urinary protein levels were significantly lower in the AR400 and AR800 groups than those in the CKD group. The results of the histomorphological analysis were shown in Fig. 2. Glomerular volume and severity of interstitial fibrosis were lower in the AR400 and AR800 groups than those in the CKD group.

**Table 1:** Animal characteristics in each group at baseline

	Sham (N = 10)	CKD (N = 10)	AR 400 (N = 11)	AR 800 (N = 12)
Body weight (g)	351 ± 8	327 ± 7	343 ± 6	346 ± 5
Blood pressure (mmHg)	118 ± 2	125 ± 2	124 ± 2	122 ± 2
Creatinine clearance (ml/min)	4.08 ± 0.38	1.21 ± 0.08*	1.17 ± 0.07*	1.31 ± 0.07*
Creatinine (mg/dL)	0.23 ± 0.02	0.79 ± 0.04*	0.85 ± 0.02*	0.85 ± 0.03*
Blood urea nitrogen (mg/dL)	21.2 ± 0.6	48.2 ± 1.1*	49.1 ± 1.0*	48.1 ± 1.2*
Albumin (g/dL)	3.4 ± 0.1	3.3 ± 0.2	3.6 ± 0.3	3.9 ± 0.1
Total cholesterol (mg/dL)	50.8 ± 3.6	65.1 ± 6.3	66.5 ± 3.3	70.3 ± 4.0
Calcium (mg/dL)	10.3 ± 0.3	10.4 ± 0.2	10.1 ± 0.2	10.1 ± 0.1
Phosphorus (mg/dL)	8.7 ± 0.4	8.5 ± 0.3	8.9 ± 0.2	9.1 ± 0.3
Glucose (mg/dL)	162 ± 8	130 ± 7	145 ± 10	147 ± 6
Urinary protein (mg/day)	16.2 ± 2.6	19.5 ± 1.1	14.5 ± 2.1	25.1 ±3.3

*P < 0.05 *vs*. the sham group

*CKD* chronic kidney disease, *AR* astragalus

**Table 2:** Animal characteristics in each group at 14 weeks of age.

	Sham (N = 10)	CKD (N = 10)	AR 400 (N = 11)	AR 800 (N = 12)
Body weight (g)	503 ± 16	478 ± 10	478 ± 8	491 ± 6
Residual kidney weight (g)	1.64 ± 0.04	2.30 ± 0.16*	2.01 ± 0.07*	1.97 ± 0.08
Heart weight (g)	1.17 ± 0.03	1.20 ± 0.08	1.15 ± 0.04	1.15 ± 0.02
Urine volume (ml/day)	29.5 ± 4.0	45.8 ± 3.3*	44.8 ± 2.1*	40.3 ± 2.0*
Urinary sodium (mEq/day)	3.11 ± 0.21	3.21 ± 1.1	3.30 ± 0.14	3.42 ± 0.13
Urinary potassium (mEq/day)	5.11 ± 0.37	4.76 ± 0.29	4.99 ± 0.24	5.23 ± 0.34
Urinary protein (mg/day)	18.9 ± 2.2	46.6 ± 10.7*	24.7 ± 4.6^#^	14.4 ± 2.2^#^

*P < 0.05 *vs*. the sham group

#P < 0.05 *vs*. the CKD group

*CKD* chronic kidney disease, *AR* astragalus

### Assessment of oxidative stress

**Fig. 3:**
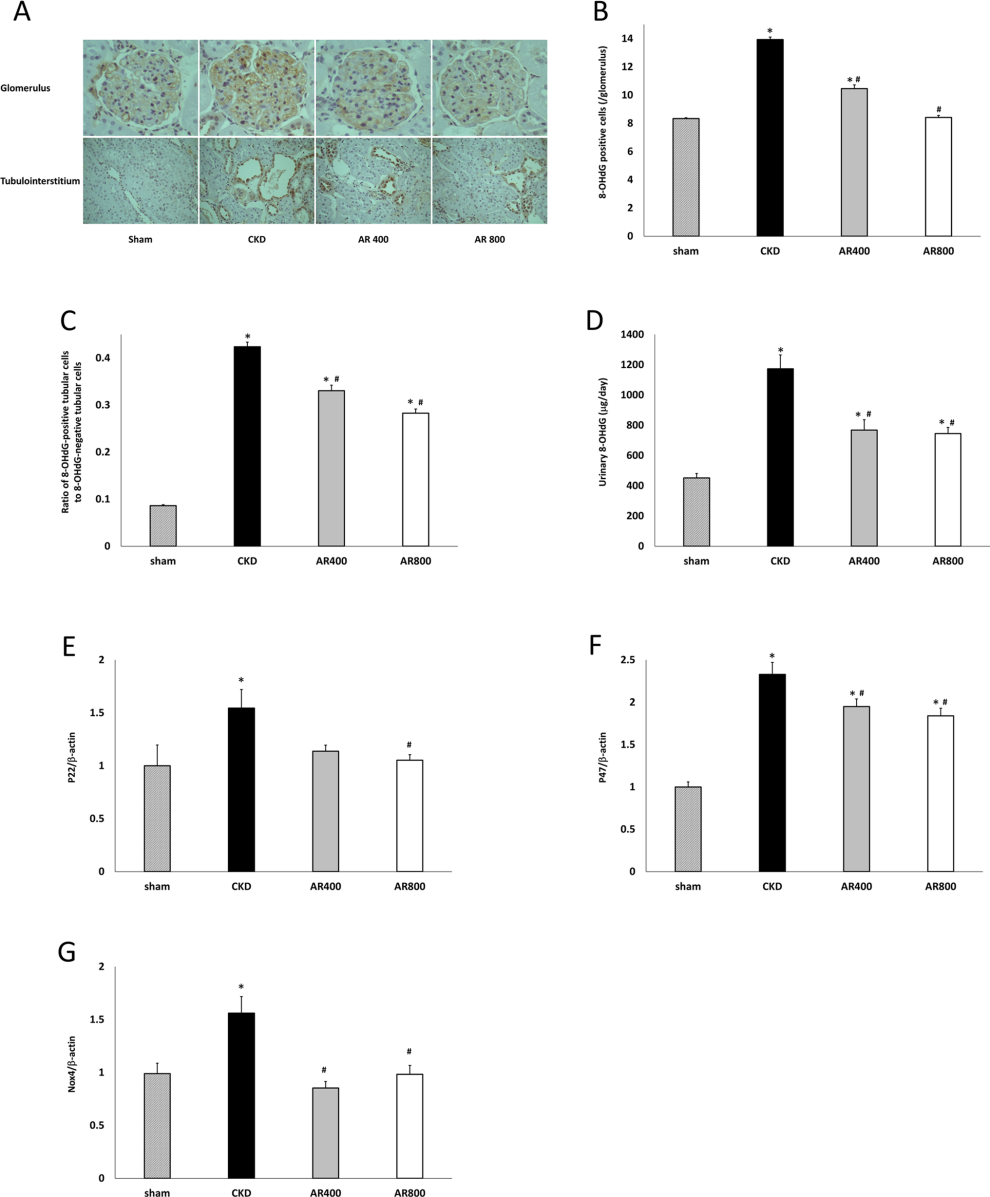
Assessment of oxidative stress. **A** Representative images of glomerulus and tubulointerstitium with immunohistochemical staining for 8-OHdG. (B) The number of 8-OHdG positive cells in glomeruli and (**C**) tubules. **D** Urinary 8-OHdG levels. **E** mRNA expression of NADPH p22 phox. **F** mRNA expression of NADPH p47 phox. **G** mRNA expression of Nox4. * P < 0.05 *vs*. the sham group, ^#^ P < 0.05 *vs*. the CKD group

The formation of 8-OHdG in glomeruli and kidney tubules increased in the CKD group, and Astragalus reduced oxidative stress induced by decreased kidney function (Fig. 3A–C). Urinary 8-OHdG excretion was greater in the CKD group than in the sham group, and those in the AR400 and AR800 groups were significantly lower than that in the CKD group (Fig. 3D). We also evaluated the mRNA expression of oxidative stress-related markers, p22 (Fig. 3E), p47 (Fig. 3F), and Nox4 (Fig. 3G) in the kidney. These mRNA expressions also increased in the CKD group. The mRNA expression of p22 in the AR800 group was significantly lower than that in the CKD group, and that in the AR400 group tended to be lower than that in the CKD group. The mRNA expressions of p47 and Nox4 were significantly lower in the AR400 and AR800 groups than in the CKD group.

### RAS assessment

**Fig. 4:**
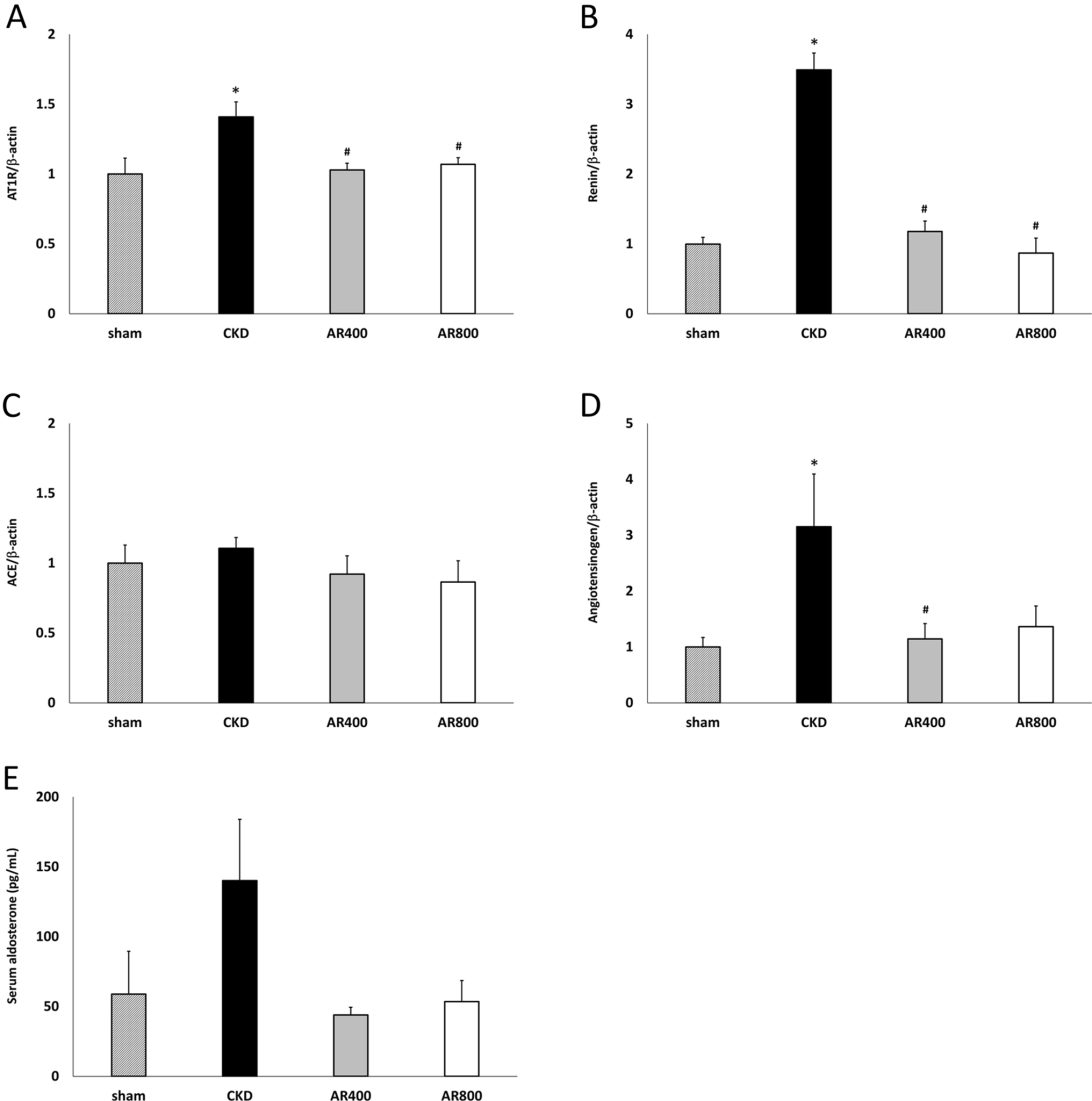
Assessment of the renin–angiotensin system in the kidney. **A** mRNA expression of renin. **B** mRNA expression of AT1R. **C** mRNA expression of ACE. **D** mRNA expression of angiotensinogen. **E** Serum aldosterone levels. *P < 0.05 *vs*. the sham group, ^#^P < 0.05 *vs*. the CKD group

To assess RAS, we measured the mRNA expressions of renin, AT1R, ACE, and angiotensinogen. The mRNA expressions of renin, AT1R, and angiotensinogen significantly increased in the CKD group than in the sham group. The mRNA expressions of renin and AT1R significantly decreased in the AR400 and AR800 groups than in the CKD group, and the mRNA expressions of angiotensinogen significantly decreased in the AR400 groups than in the CKD group. The mRNA expressions of ACE were comparable among groups (Fig. 4A–D). Although serum aldosterone levels in the AR400 and AR800 groups tended to be lower than those in the CKD groups, the difference was not statistically significant (Fig. 4E).

## Discussion

Our study demonstrated that astragalus root attenuated the deterioration of kidney function, decreased proteinuria, and suppressed the rise of blood pressure in 5/6 nephrectomized rats. Furthermore, it decreased urinary excretion of oxidative stress marker 8-OHdG levels and suppressed the increased expression of oxidative stress and RAS in kidney tissue.

Some clinical and animal studies have shown the renoprotective effects of the astragalus root. Two meta-analysis studies on its renoprotective effects have been reported in clinical settings [5, 24]. One meta-analysis study demonstrated that pooled results in 13 randomized control trials indicated that astragalus significantly decreased 21.39 [95% confidence interval (CI) 8.00, 34.78] µmlo/L (0.24 [95% CI 0.09, 0.39] mg/dL) of serum creatinine levels and pooled results in 10 randomized control trials indicated that astragalus significantly decreased 0.53 [95% CI 0.26, 0.79] g/day of proteinuria compared with control [5]. The other meta-analysis study targeted at diabetic kidney disease also revealed a decrease in serum creatinine levels and proteinuria by the additional use of astragalus [24]. In animal studies, astragalus attenuated kidney injury and reduced proteinuria in various models, including 5/6 nephrectomy, glomerulonephritis, unilateral ureteral obstruction, doxorubicin-induced nephropathy and streptozotocin-induced diabetic nephropathy [6]. Corresponding with these findings, astragalus root attenuated the decrease in creatinine clearance and reduced urinary protein in our study.

Astragalus root may have beneficial effects on both glomeruli and tubules. Astragaloside IV, one of the active components of astragalus, was reported to attenuate podocyte injury [25]. In addition, another study has shown that astragalus and angelica reduced glomerular injury in kidney tissue [16]. On the other hand, astragalus has also improved proximal tubular cell viability [26] and reduced tubulointerstitial injury in kidney tissue [16]. In our study, both glomerular damage marker urinary albumin and tubular damage marker urinary NGAL in the astragalus groups were significantly lower than those in the CKD group. Therefore, the astragalus root may have a beneficial effect on both glomeruli and tubules.

Oxidative stress contributes to the deterioration of kidney function [8]. Several studies have shown the anti-oxidative effects of astragalus. For example, in an experimental study using human kidney proximal tubular epithelial cells, astragalus reduced cell apoptosis induced by hydrogen peroxide, a precursor to harmful reactive oxygen species [26]. In another study using CKD mouse models, astragalus reduced oxidative stress in kidney tissue induced by heminephrectomy and indoxyl sulfate [27]. Our study also demonstrated that astragalus decreased urinary excretion of 8-OHdG levels and reduced the expression of several oxidative stress markers in kidney tissue. Therefore, these findings indicate that astragalus has anti-oxidative effects for chronic kidney disease.

The effect of astragalus on RAS is controversial [14–18]. An in vitro study using mouse serum indicated that an aqueous extract of astragalus root dose-dependently inhibited ACE [14]. This study also showed that one of the peptide fragments included in the extract of astragalus interacted with active residues of the ACE. Total flavonoid extracted from astragalus seed has been reported to decrease plasma angiotensin II levels in hypertensive rats [15]. Our study indicated that astragalus suppressed RAS-related markers expressions in kidney tissue with decreased renal function. Since RAS blockers reduced the risk of kidney failure [13], astragalus may have the same favorable effect by inhibiting RAS.

Our study showed that astragalus decreased blood pressure elevation during the course of CKD. This anti-hypertensive effect of astragalus has been shown in spontaneously hypertensive rats, heminephrectomy/deoxycorticosterone acetate/salt-induced hypertensive mice, and angiotensin II-induced hypertensive mice [14, 28]. Considering the finding that astragalus has an inhibitory effect on RAS, its anti-hypertensive effects may be due to RAS inhibition.

Astragalus includes various components. Among these components, it is not completely elucidated which components are the main factors to attenuate kidney damage. As we mentioned above, one peptide included in astragalus has been reported to have potential ACE inhibitor activities and interact with active residues of ACE [14]. Therefore, the peptide may be one of the factors to attenuate kidney damage. Astragaloside IV, which is one of the major substances of astragalus, has the potential effect of organ fibrosis, inflammatory response, oxidative stress, and apoptosis [29]. Since the substance has been demonstrated to attenuate kidney injury induced by indoxyl sulfate [27] and restore podocyte morphology and cytoskeleton loss [25], astragaloside IV may also be one of the renoprotective factors. Furthermore, the report to explore the proteins related to the effect of astragalus on diabetic nephropathy using network pharmacology has shown that quercetin, formononetin, calycosin, 7-O-methylisomucronultol, and quercetin are the potential factors related to the therapeutic effect of astragalus [30]. Taken together, although some components are suggested as the main renoprotective factors of astragalus, further studies are necessary to reveal the factors.

In conclusion, our findings indicate that astragalus has renoprotective and anti-hypertensive effects in a CKD rat model, which could be attributed to the suppression of oxidative stress and RAS.

## Data Availability

The data that support the findings of this study are available from the corresponding author, H.F, upon reasonable request.

## References

[1] Hill NR, Fatoba ST, Oke JL, Hirst JA, O'Callaghan CA, Lasserson DS, Hobbs FD (2016). Global prevalence of chronic kidney disease - a systematic review and meta-analysis. PLoS ONE.

[2] Matsushita K, van der Velde M, Astor BC, Woodward M, Levey AS, de Jong PE, Coresh J, Chronic Kidney Disease Prognosis Consortium (2010). Gansevoort RT. association of estimated glomerular filtration rate and albuminuria with all-cause and cardiovascular mortality in general population cohorts: a collaborative meta-analysis. Lancet.

[3] Fukuhara S, Lopes AA, Bragg-Gresham JL, Kurokawa K, Mapes DL, Akizawa T, Bommer J, Canaud BJ, Port FK, Held PJ (2003). Worldwide dialysis outcomes and practice patterns study. health-related quality of life among dialysis patients on three continents: the dialysis outcomes and practice patterns study. Kidney Int.

[4] Nitta K, Goto S, Masakane I, Hanafusa N, Tanguchi M, Hasegawa T, Nakai S, Wada A, Hamano T, Hoshino J, Joki N, Abe M, Yamamoto K, Nakamoto H (2020). Annual dialysis data report for 2018, JSDT Renal data registry: survey methods, facility data, incidence, prevalence, and mortality. Ren Replace Ther.

[5] Zhang HW, Lin ZX, Xu C, Leung C, Chan LS. Astragalus (a traditional Chinese medicine) for treating chronic kidney disease. Cochrane Database Syst Rev 10: CD008369. (2014) 10.1002/14651858.CD008369.pub2 PMC10589061 25335553

[6] Zhong Y, Deng Y, Chen Y, Chuang PY, Cijiang HJ (2013). Therapeutic use of traditional Chinese herbal medications for chronic kidney diseases. Kidney Int.

[7] Yoshino T, Horiba Y, Mimura M, Watanabe K (2022). Oral Astragalus root supplementation for mild to moderate chronic kidney disease: a self-controlled case-series. Front Pharmacol.

[8] Verma S, Singh P, Khurana S, Ganguly NK, Kukreti R, Saso L, Rana DS, Taneja V, Bhargava V (2021). Implications of oxidative stress in chronic kidney disease: a review on current concepts and therapies. Kidney Res Clin Pract.

[9] Fujii H, Yonekura Y, Yamashita Y, Kono K, Nakai K, Goto S, Sugano M, Goto S, Fujieda A, Ito Y, Nishi S (2016). Anti-oxidative effect of AST-120 on kidney injury after myocardial infarction. Br J Pharmacol.

[10] Yonekura Y, Fujii H, Nakai K, Kono K, Goto S, Shinohara M, Nishi S (2015). Anti-oxidative effect of the beta-blocker carvedilol on diabetic nephropathy in non-obese type 2 diabetic rats. Clin Exp Pharmacol Physiol.

[11] Nakai K, Fujii H, Kono K, Goto S, Kitazawa R, Kitazawa S, Hirata M, Shinohara M, Fukagawa M, Nishi S (2014). Vitamin D activates the Nrf2-Keap1 antioxidant pathway and ameliorates nephropathy in diabetic rats. Am J Hypertens.

[12] Fujii H, Kono K, Nakai K, Goto S, Komaba H, Hamada Y, Shinohara M, Kitazawa R, Kitazawa S, Fukagawa M (2010). Oxidative and nitrosative stress and progression of diabetic nephropathy in type 2 diabetes. Am J Nephrol.

[13] Kidney Disease: Improving Global Outcomes (KDIGO) Blood Pressure Work Group. KDIGO (2021). Clinical practice guideline for the management of blood pressure in chronic kidney disease. Kidney Int.

[14] Wu JS, Li JM, Lo HY, Hsiang CY, Ho TY (2020). Anti-hypertensive and angiotensin-converting enzyme inhibitory effects of Radix Astragali and its bioactive peptide AM-1. J Ethnopharmacol.

[15] Li JX, Xue B, Chai Q, Liu ZX, Zhao AP, Chen LB (2005). Antihypertensive effect of total flavonoid fraction of Astragalus complanatus in hypertensive rats. Chin J Physiol.

[16] Wang H, Li J, Yu L, Zhao Y, Ding W (2004). Antifibrotic effect of the Chinese herbs, Astragalus mongholicus and Angelica sinensis, in a rat model of chronic puromycin aminonucleoside nephrosis. Life Sci.

[17] Zhang YW, Xie D, Xia B, Zhen RT, Liu IM, Cheng JT (2006). Suppression of transforming growth factor-beta1 gene expression by Danggui buxue tang, a traditional Chinese herbal preparation, in retarding the progress of renal damage in streptozotocin-induced diabetic rats. Horm Metab Res.

[18] Hou G, Jiang Y, Zheng Y, Zhao M, Chen Y, Ren Y, Wang C, Li W (2021). Mechanism of radix astragali and radix salviae miltiorrhizae ameliorates hypertensive renal damage. Biomed Res Int.

[19] Reagan-Shaw S, Nihal M, Ahmad N (2008). Dose translation from animal to human studies revisited. FASEB J.

[20] Okuda M, Horikoshi S, Matsumoto M, Tanimoto M, Yasui H, Tomino Y (2012). Beneficial effect of Astragalus membranaceus on estimated glomerular filtration rate in patients with progressive chronic kidney disease. Hong Kong J Nephrol.

[21] Ichinose K, Maeshima Y, Yamamoto Y, Kitayama H, Takazawa Y, Hirokoshi K, Sugiyama H, Yamasaki Y, Eguchi K, Makino H (2005). Antiangiogenic endostatin peptide ameliorates renal alterations in the early stage of a type 1 diabetic nephropathy model. Diabetes.

[22] Yonekura Y, Fujii H, Nakai K, Kono K, Goto S, Shinohara M, Nishi S (2015). Anti-oxidative effect of the β-blocker carvedilol on diabetic nephropathy in non-obese type 2 diabetic rats. Clin Exp Pharmacol Physiol.

[23] Gao X, Huang L, Grosjean F, Esposito V, Wu J, Fu L, Hu H, Tan J, He C, Gray S, Jain MK, Zheng F, Mei C (2011). Low-protein diet supplemented with ketoacids reduces the severity of renal disease in 5/6 nephrectomized rats: a role for KLF15. Kidney Int.

[24] Zhang L, Shergis JL, Yang L, Zhang AL, Guo X, Zhang L, Zhou S, Zeng L, Mao W, Xue CC (2019). Astragalus membranaceus (Huang Qi) as adjunctive therapy for diabetic kidney disease: an updated systematic review and meta-analysis. J Ethnopharmacol.

[25] Zheng R, Deng Y, Chen Y, Fan J, Zhang M, Zhong Y, Zhu R, Wang L (2012). Astragaloside IV attenuates complement membranous attack complex induced podocyte injury through the MAPK pathway. Phytother Res.

[26] Shahzad M, Small DM, Morais C, Wojcikowski K, Shabbir A, Gobe GC (2016). Protection against oxidative stress-induced apoptosis in kidney epithelium by Angelica and Astragalus. J Ethnopharmacol.

[27] Ji C, Luo Y, Zou C, Huang L, Tian R, Lu Z (2018). Effect of astragaloside IV on indoxyl sulfate-induced kidney injury in mice via attenuation of oxidative stress. BMC Pharmacol Toxicol.

[28] Zheng W, Huang T, Tang QZ, Li S, Qin J, Chen F (2021). Astragalus polysaccharide reduces blood pressure, renal damage, and dysfunction through the TGF-β1-ILK pathway. Front Pharmacol.

[29] Li L, Hou X, Xu R, Liu C, Tu M (2017). Research review on the pharmacological effects of astragaloside IV. Fundam Clin Pharmacol.

[30] Guo MF, Dai YJ, Gao JR, Chen PJ (2020). Uncovering the mechanism of astragalus membranaceus in the treatment of diabetic nephropathy based on network pharmacology. J Diabetes Res.

